# Analyzing large-scale samples confirms the association between the rs1051730 polymorphism and lung cancer susceptibility

**DOI:** 10.1038/srep15642

**Published:** 2015-10-28

**Authors:** Zhijie Han, Qinghua Jiang, Tianjiao Zhang, Xiaoliang Wu, Rui Ma, Jixuan Wang, Yang Bai, Rongjie Wang, Renjie Tan, Yadong Wang

**Affiliations:** 1 School of Life Science and Technology, Harbin Institute of Technology, Harbin, 150001, China; 2 School of Computer Science and Technology, Harbin Institute of Technology, Harbin, 150001, China; 3 School of Software, Harbin Institute of Technology, Harbin, 150001, China

## Abstract

The early genome-wide association studies (GWAS) found a significant association between lung cancer and rs1051730 (15q25) polymorphism. However, the subsequent studies reported consistent and inconsistent results in different populations. Three meta-analysis studies were thus performed to reevaluate the association. But their results remain inconsistent. After that, some new GWAS studies reported conflicting results again. We think that the divergence of these results may be due to small-scale samples or heterogeneity among different populations. Therefore, we reevaluated the association by collecting more samples (N = 33,617 cases and 116,639 controls) from 31 studies, which incorporate 8 new studies and 23 previous studies used by one or more of the three meta-analysis studies. We observed a significant association between lung cancer and rs1051730 in pooled population by using allele (OR = 1.30, 95% CI = 1.27–1.34, P  <  0.0001), dominant (OR = 1.41, 95% CI = 1.29–1.55, P < 0.0001), recessive (OR = 1.53, 95% CI = 1.42–1.65, P < 0.0001) and additive (OR = 1.75, 95% CI = 1.61–1.90, P < 0.0001) models. Through the subgroup analysis, we observed a significant heterogeneity only in East Asian population (P = 0.006, I^2^ = 66.9%), and the association is significant in all subgroups (OR = 1.2976, 95% CI = 1.2622–1.3339 (European ancestry), OR = 1.5025, 95% CI = 1.2465–1.8110 (African), OR = 1.7818, 95% CI = 1.3915–2.2815 (East Asian), P < 0.0001). We believe that these results will contribute to understanding the genetic mechanism of lung cancer.

Lung cancer is one of the most dangerous diseases, and the morbidity and mortality are increasing constantly around the world today^1^. According to the statistics from American Cancer Society (ACS), the new cases of lung cancer were 0.221 million and dead 0.158 million in the United States in 2015, which was one of the most frequently diagnosed cancers and the leading causes of cancer death in men and women^2^. Even though, the majority of studies proved that over 80 percent lung cancer occurrence is associated with the habit of smoking, while only 20 percent of these smokers will develop into lung cancer, which suggests that lung cancer occurrence possesses genetic susceptibility^1^,^3^.

Many single nucleotide polymorphism (SNP) sites in association with lung cancer were obtained by the genome–wide association studies (GWAS)^4^. For example, Amos *et al*.^5^, Thorgeirsson, *et al*.^6^ and Shiraishi *et al*.^7^ found that the rs1051730 SNP was associated with lung cancer in European ancestry and Japanese population, respectively. Amos *et al*. analyzed 956 cases and 1,830 controls from the UK, and discovered the odds ratio (OR) = 1.32, 95% confidence interval (CI) is from 1.23 to 1.39, and P < 10E–08. Thorgeirsson, *et al*. analyzed 665 cases and 28,752 controls from the Iceland, 269 cases and 1,474 controls from the Spain, 90 cases and 2,018 controls from the Netherlands, and the combined result showed that OR = 1.31, 95% CI is from 1.19 to 1.44, and P = 1.5E–08. Shiraishi *et al*. analyzed 1,250 cases and 936 controls from the Japan, and found the OR = 2.3, 95% CI is from 1.5 to 3.7, and P = 0.0028.

However, the subsequent studies reported consistent^8^,^9^,^10^,^11^,^12^,^13^,^14^,^15^,^16^,^17^,^18^ and inconsistent^19^,^20^ results. For example, Wu *et al*.^19^ used 1,151 cases and 1,150 controls from China, and Girard *et al*.^20^ used 94 cases and 95 controls from America to investigate the association between rs1051730 and lung cancer, and obtained negligible or weak association.

Facing the inconsistent results above, Gu *et al*.^21^, Hu *et al*.^22^ and Zhan *et al*.^23^ collected different samples to re-assess the association between rs1051730 and lung cancer by meta-analysis, respectively. However, the results of the three meta-analysis studies are also inconsistent. Gu *et al*.^21^ and Hu *et al*.^22^ found that the association between rs1051730 and lung cancer is not significant, while it is significant according to the study of Zhan *et al*.^23^.

After these studies, five articles^24^,^25^,^26^,^27^,^28^, which include eight studies, investigated the association between rs1051730 and lung cancer. But the results of eight studies remain inconsistent. For example, He *et al*.^27^ used 301 cases and 318 controls from China, Hansen *et al*.^28^ used 448 cases and 611 controls from America, and found a negligible association between rs1051730 and lung cancer, while other researches^24^,^25^,^26^ reported opposite consequences.

We considered that the divergence of these results may be due to small-scale samples or heterogeneity among different populations. Here, we collected all the samples of the three meta-analysis studies and the eight studies from the five new articles, and thus obtained a larger sample sizes (33,617 cases and 116,639 controls) from 31 studies to reevaluate the association between rs1051730 polymorphism and lung cancer based on the method that was frequently used by Liu *et al*. to study Alzheimer’s Disease^29^,^30^,^31^,^32^ and colorectal cancer^33^.

## Results

### Literature and Study acquisition as well as Data extraction

**Figure 1:**
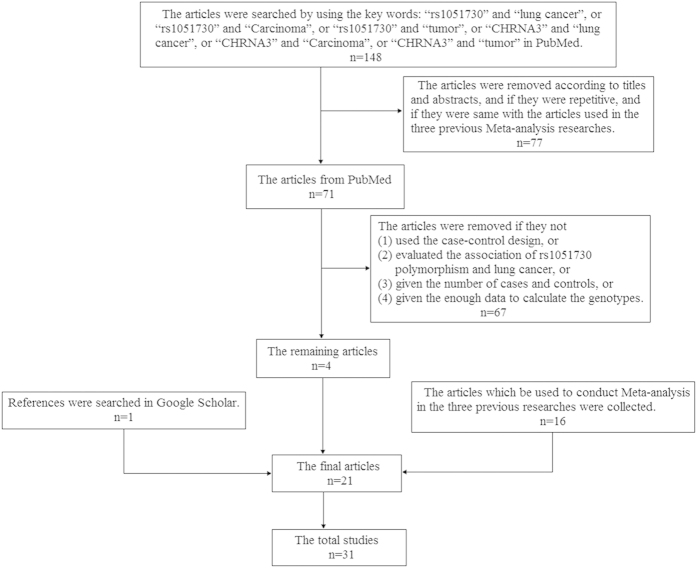
The flow chart of collecting articles for analyzing the association between rs1051730 polymorphism and lung cancer. The criteria of selecting articles are: (1) the study was designed according to the method of case-control. (2) the study evaluated the association of rs1051730 polymorphism and lung cancer. (3) the number of cases and controls was provided in the study. (4) the study provided the population of each individual. (5) the study provided the number of rs1051730 genotypes both in cases and controls or provided enough data to calculate the genotypes. (6) the study provided the OR value with 95% CI and the P value or provided enough data to calculate them.

By searching PubMed with keywords (details shown in the method section), we obtained 20 articles, which include 16 articles used in the three previous meta-analysis researches and 4 new articles. Moreover, we obtained another article through the reference search in Google Scholar. Finally, we got 31 studies from the 21 articles according to the six inclusion criteria (details shown in the method section). The workflow was showed in Fig. 1. After that, we collected the relative data according to 11 terms for each study, and 7 of the 11 terms were listed in Table 1.

### Heterogeneity Test

According to the kind of genotype shown in Table 1, we used 30 studies in the allele model, 17 studies in the dominant model, 14 studies in the recessive model, and 13 studies in the additive model, respectively. We didn’t get a significant heterogeneity in the allele model (P = 0.0161 and I^2^ = 39.1%), recessive model (P = 0.4208 and I^2^ = 2.7%), and additive model (P = 0.1791 and I^2^ = 26.3%). However, it is significant in the dominant model (P = 0.0023 and I^2^ = 56.4%).

### Meta-analysis

**Figure 2:**
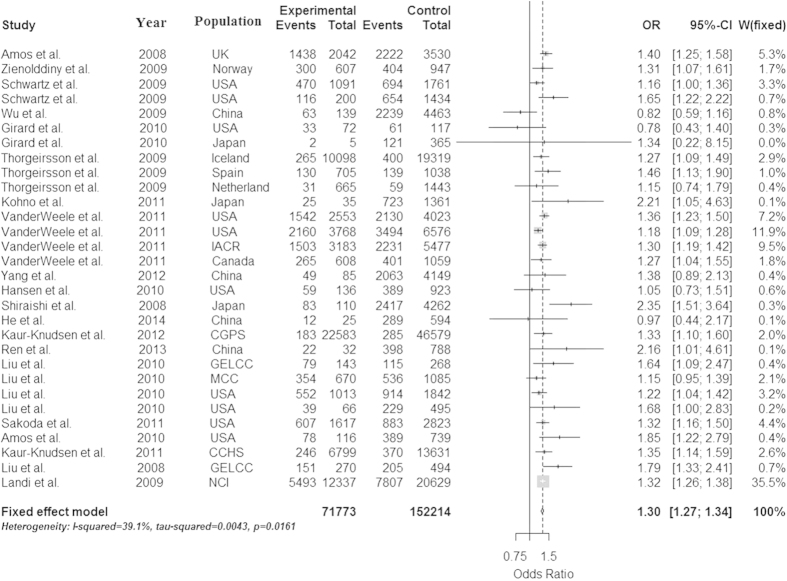
Forest plot for the meta-analysis of rs1051730 polymorphism using allele model. According to Table 1, 30 studies that provided the data of genotype A/G and AA/GA/GG and GA/GG were used in the allele model (A vs G).

**Figure 3:**
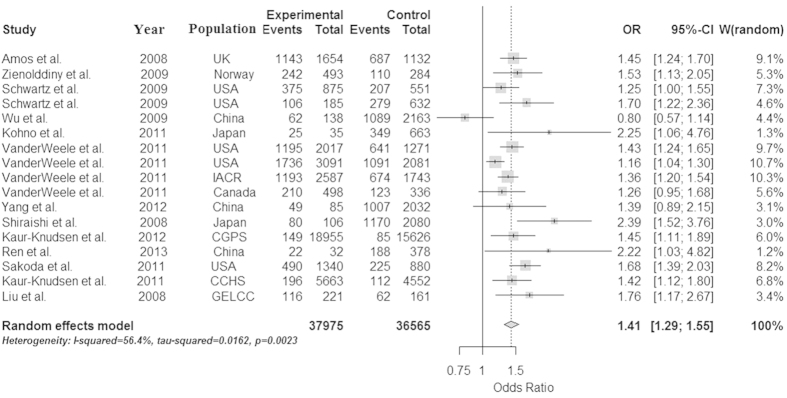
Forest plot for the meta-analysis of rs1051730 polymorphism using dominant model. According to Table 1, 17 studies that provided the data of genotype AA/GA/GG and GA/GG were used in the dominant model (AA+AG vs GG).

**Figure 4:**
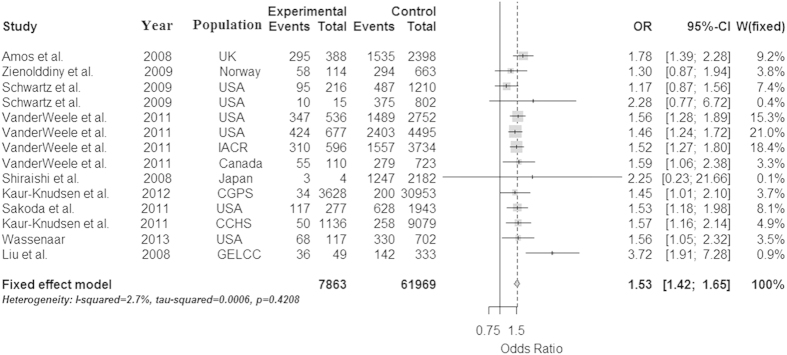
Forest plot for the meta-analysis of rs1051730 polymorphism using recessive model. According to Table 1, 14 studies that provided the data of genotype AA/GA/GG as well as AA/GA and GG were used in the recessive model (AA vs AG+GG).

**Figure 5:**
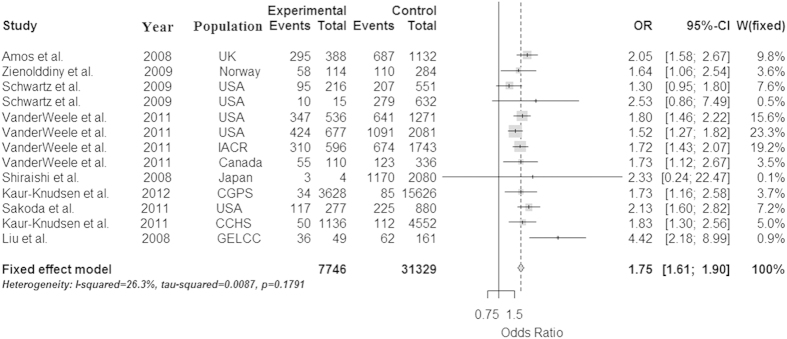
Forest plot for the meta-analysis of rs1051730 polymorphism using additive model. According to Table 1, 13 studies that provided the data of genotype AA/GA/GG were used in the additive model (AA vs GG).

Because the heterogeneity is significant in the dominant model, we used the random effect model to analyze it. And because the heterogeneity isn’t significant in the allele, recessive and additive models, we used the fix effect model to analyze them, and get a relatively strong association between the rs1051730 and lung cancer in the four models (OR = 1.30 and 95% CI = 1.27–1.34 in the allele model, OR = 1.41 and 95% CI = 1.29–1.55 in the dominant model, OR = 1.53 and 95% CI = 1.42–1.65 in the recessive model, OR = 1.75 and 95% CI = 1.61–1.90 in additive model, respectively). The P values of Z test are less than 0.0001 in all models, which proved that these OR values are believable. These conclusions were described in Figs 2, 3, 4, 5.

### Subgroup Analysis

Because the allele model included the maximum number of studies, we further used it to perform the subgroup analysis. We found that there is no significant heterogeneity in European ancestry (P = 0.2296 and I^2^ = 18.4%) and African (P = 0.1586 and I^2^ = 42.2%) populations, while in East Asian population the heterogeneity is significant (P = 0.006 and I^2^ = 66.9%). So we further split East Asian population into Japanese and Chinese subgroups. The heterogeneity was not found in the Japanese population (P = 0.8387 and I^2^ = 0), but it remains significant in the Chinese population (P = 0.0753 and I^2^ = 56.5%). Then, we removed each study from Chinese population orderly, and found that there was no significant heterogeneity after the study of Wu *et al*. had been removed. After meta-analysis and Z test, we found the association between the rs1051730 and lung cancer is relatively strong in all populations. The results were described in Table 2. Forest plots of each subgroup meta-analysis were shown in Figure S1–S7.

### Publication Bias Analysis and Sensitivity Analysis

**Figure 6:**
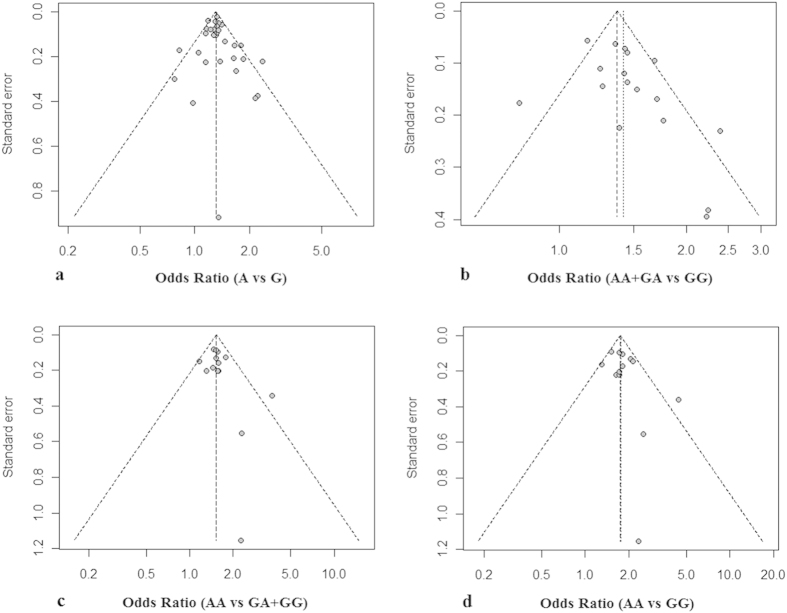
Funnel plot of publication bias analysis in the allele, dominant, recessive and additive model. This funnel plot shows the result of the publication bias analysis for rs1051730 polymorphism with lung cancer using allele (**a**), dominant (**b**), recessive (**c**) and additive (**d**) model. The X-axis represents OR value and the Y-axis represents standard error of each study, respectively.

We used Begg’s test and Egger’s test to measure the publication bias for the allele, dominant, recessive and additive models, and didn’t find a significant publication bias in all these models. The P values of Begg’s test and Egger’s test for the allele, dominant, recessive and additive models are 0.4077 and 0.3897, 0.1061 and 0.0869, 0.1838 and 0.2044, 0.1314 and 0.1619, respectively. The funnel plot (Fig. 6) reflected the result directly. And then, the result of sensitivity analysis showed that the association between rs1051730 and lung cancer doesn’t extremely change when we removed each of the studies in the four models orderly. The detail information was in supplement materials (Table S5–S8).

## Discussion

Nicotine receptor protein abnormal expression is one of the reasons for lung cancer occurrence^3^. CHRNA3, a gene coding a part of nicotinic acetylcholine receptor protein subunits, includes a SNP rs1051730. The elder GWAS studies showed that a significant association between the rs1051730 polymorphism and lung cancer in the European ancestry and Japanese population^5^,^6^,^7^. However, the subsequent studies that researched the association in various populations or in different scales reported consistent and inconsistent results. We think the divergence of these results might be due to the small-scale samples or heterogeneity among different populations. So we summarized these studies in a larger scale and in a more comprehensive population and found that the association between rs1051730 polymorphism and lung cancer is significant by using the allele model (OR = 1.30, 95% CI = 1.27–1.34, P < 0.0001), dominant model (OR = 1.41, 95% CI = 1.29–1.55, P < 0.0001), recessive model (OR = 1.53, 95% CI = 1.42–1.65, P < 0.0001) and additive model (OR = 1.75, 95% CI = 1.61–1.90, P < 0.0001). The farther subgroup analysis showed a significant association in European ancestry (OR = 1.2976, 95% CI = 1.2622–1.3339, P < 0.0001) and African (OR = 1.5025, 95% CI = 1.2465–1.8110, P < 0.0001) populations. But in East Asian population, we found a weak association with a non–significant result of Z test (OR = 1.4759, CI = 0.9957–2.1878, P = 0.0526), and the heterogeneity is significant (P = 0.006 and I^2^ = 66.9%). So we further split East Asian population into Japanese and Chinese subgroups. The result indicated that there is a significant association in the Japanese population (OR = 2.2654, 95% CI = 1.5675–3.2741, P < 0.0001), while the result of Z test is still non-significant in the Chinese population (P = 0.4389). We removed each study from Chinese population orderly, and found a significant association (OR = 1.4235, 95% CI = 1.0146–1.9972, P = 0.041) after the study of Wu *et al*. had been removed. We think this phenomenon may be caused by a relatively small sample size in the study of Wu *et al*. Moreover, we removed the study of Wu *et al* from East Asian population, and result of meta-analysis showed that the association is significant in East Asian population (OR = 1.7818, 95% CI = 1.3915–2.2815, P < 0.0001). Consequently, the association between rs1051730 polymorphism and lung cancer is significant in all of these populations. In addition, the result of sensitivity analysis reflects the conclusion is robust, and the publication bias isn’t significant.

Before submitting this paper, we used keyword “rs1051730”, “lung cancer” and “meta” to search in PubMed, and obtained six articles^21^,^22^,^23^,^34^,^35^,^36^, which include the researches of Gu *et al*.^21^, Hu *et al*.^22^ and Zhan *et al*.^23^. Among these three articles, Gu *et al*.^21^ integrated 16 studies to assess the risk of rs1051730 in East Asian, European, and African populations by using allelic and dominant models. Hu *et al*.^22^ also collected 16 studies in the same populations, but merely the allelic model was used. They obtained a similar result that the risk is high in European and African populations, but it is weak in East Asian. Zhan *et al*.^23^ only assessed the East Asian, they combined 4 studies and the result shows a significant association between the rs1051730 and lung cancer. The other three articles didn’t research the association between the rs1051730 polymorphism and lung cancer. They evaluated the association between rs1051730 and habit of smoking^34^,^35^,^36^, or the association between rs1051730 and cotinine levels^36^ by meta-analysis.

Our work is different from the others. We collected all 31 studies, which include 23 studies used by the three previous meta-analysis researches and 8 new studies. We analyzed the association between rs1051730 and lung cancer in European ancestry, African and East Asian populations. The association is significant in European ancestry and African populations, which is consistent with the result of Gu *et al*.^21^ and Hu *et al*.^22^. However, we found that the association remains significant in East Asian population, which is not consistent with the result of Gu *et al*.^21^ and Hu *et al*.^22^.

The rs1051730 polymorphism is in CHRNA3 on 15q25. CHRNA3 is one of members in a multigene family of nicotinic acetylcholine receptor (nAChR gene cluster) which can code various nicotinic acetylcholine receptor protein subunits include: α3, α4, α7, α9, α10, β2 and β4 nAChR subunits^37^. These subunits are expressed on the bronchial epithelial cells of human being and primates^38^. Through combining with the nicotinic acetylcholine receptor protein, nicotine promotes tumor cell proliferation, invasion, migration and induces blood vessel formation. At the same time, it provides a protection for the tumor cell to avoid the programmed cell death^39^. In addition, Arredondo *et al*.^40^ found that α3, α4, α7, α9, α10, β2 and β4 nAChR subunits can form the high-affinity sites to bind 4-(methylnitro-samino)-1-(3-pyridyl)-1-butanone (NNK), a cancerogen produced through nicotine nitrosylation, thus to increase the risk of lung cancer. Moreover, CHRNA3 also contains other two SNP: rs578776 and rs938682 polymorphism. The rs17486278, rs11637635 as well as rs7178270 polymorphism belong to CHRNA5 and CHRNA4, respectively. They may be also associated with the susceptibility of non-small cell lung cancer was reported in a study^41^. In addition, another gene AGPHD1 also in 15q25 contains the rs8034191 polymorphism, and many studies reported its association with lung cancer^5^,^8^,^9^,^20^,^24^. We expect that more research on them could be performed in the future.

## Methods

### Literature and Study acquisition

We collected all the articles which were used to perform meta-analysis by Gu *et al*.^21^, Hu *et al*.^22^ and Zhan *et al*.^23^. And then, we searched all the possible articles in PubMed (http://www.ncbi.nlm.nih.gov/pubmed) with keywords: “rs1051730” and “lung cancer”, or “rs1051730” and “Carcinoma”, or “rs1051730” and “tumor”, or “CHRNA3” and “lung cancer”, or “CHRNA3” and “Carcinoma”, or “CHRNA3” and “tumor”. All of these literatures had been collected before the PubMed’s last update on April 7 2015. In addition, we selected the related references in these articles both from PubMed and the three meta-analysis researches by using Google Scholar (http://scholar.google.com/). All the selected articles were written in English.

After that, we selected the studies in all obtained articles according to the following criteria: (1) The study was designed according to the method of case-control. (2) The study evaluated the association of rs1051730 polymorphism and lung cancer. (3) The number of cases and controls was provided in the study. (4) The study provided the population of each individual. (5) The study provided the number of rs1051730 genotypes both in cases and controls or provided enough data to calculate the genotypes. (6) The study provided the OR value with 95% CI and the P value or provided enough data to calculate these.

### Data extraction

We extracted the following information from each study we have selected: (1) The first author of each article. (2) The publication year of each article. (3) The population and ethnicity of individual in each study. (4) The number of cases and controls in each study. (5) The number of rs1051730 genotypes both in cases and controls. (6) The OR value with 95% CI and the P value in each study. (7) The genotyping platform. If the information didn’t be provided directly, we used program R (http://www.r-project.org/) to work them out.

### Choice of genetic model

The rs1051730 polymorphism has two alleles A and G. Among them, A is the minor allele. We analyzed the association between rs1051730 polymorphism and lung cancer by using the allele model (A allele versus G allele), the dominant model (AA+GA versus GG), the recessive model (AA versus GA+GG), and the additive model (AA versus GG), respectively. According to Table 1, among these analyses, the studies that provided the kind of genotype A/G and AA/GA/GG and GA/GG were used in the allele model, the studies that provided the kind of genotype AA/GA/GG and GA/GG were used in the dominant model, the studies that provided the kind of genotype AA/GA/GG as well as AA/GA and GG were used in the recessive model, the studies that provided the kind of genotype AA/GA/GG were used in the additive model. These classification data according to genotype were shown in supplement materials (Table S1–S4).

### Heterogeneity Test

We used two quantities, Cochran’s Q and I^2^, to measure the heterogeneity among the different ethnic groups. Q approximately follows a χ^2^ distribution with k-1 degrees of freedom (where k is the number of studies), and the P value can be used to measure the significance level of heterogeneity. I^2^ = (Q-(K-1))/Q*100%, which ranges from 0 to 100%. Usually, we would tentatively assign adjectives of low, moderate, and high to I^2^ values of 25%, 50%, and 75%^42^. In our study, we deemed that the different ethnic groups have significant heterogeneity when P < 0.01 and I^2^ > 50%^29^,^30^,^31^,^32^,^33^.

### Meta-analysis and Subgroup Analysis

Two models can be used in meta-analysis: the random effect model and the fix effect model^43^. After heterogeneity test, we used the software package meta (http://cran.r-project.org/web/packages/meta/index.html) to perform the meta-analysis with the random effect model, if the heterogeneity is significant, and used the fix effect model, if the heterogeneity is not. And then, we used Z test to examine the significance of meta-analysis. We further conducted a subgroup analysis after meta-analysis. We split original studies into smaller groups according to population, and conducted the meta-analysis to each subgroup respectively.

### Publication Bias Analysis and Sensitivity Analysis

Begg’s test^44^ and Egger’s test^45^ are two methods to evaluate the publication bias. When the P values are less than 0.05, publication bias is deemed significant. We measured the publication bias of each group, which include the allele model, the dominant model, the recessive model and the additive model, by the Begg’s and Egger’s test. Meanwhile, we drew the funnel plot to show the bias of each group directly. And then, we removed each study in these groups orderly to measure the influence of each study. We used the software package named meta of program R for all these calculations.

## Additional Information

**How to cite this article**: Han, Z. *et al*. Analyzing large-scale samples confirms the association between the rs1051730 polymorphism and lung cancer susceptibility. *Sci. Rep*. **5**, 15642; doi: 10.1038/srep15642 (2015).

## Supplementary Material

Supplementary Information

## Figures and Tables

**Table 1:** Main information of these studies used to analyze the association between rs1051730polymorphism and lung cancer.

Study	Year	Country or Institution	Ethnicity	No. of cases	No. of controls	Genotyping platform	Kind of genotype
Amos *et al*.^5^	2008	UK	European ancestry	956	1830	PCR	AA/GA/GG
Zienolddiny *et al*.^8^	2009	Norway	European ancestry	352	425	TaqMan	AA/GA/GG
Schwartz *et al*.^9^	2009	USA	European ancestry	582	833	TaqMan	AA/GA/GG
Schwartz *et al*.^9^	2009	USA	African	385	432	TaqMan	AA/GA/GG
Wu *et al*.^19^	2009	China	East Asian	1151	1150	PCR	AA/GA/GG
Girard *et al*.^20^	2010	USA	European ancestry	94	95	Illumina 610	A/G
Girard *et al*.^20^	2010	Japan	East Asian	123	247	Illumina 610	A/G
Thorgeirsson *et al*.^6^	2008	Iceland	European ancestry	665	28752	Illumina 300	A/G
Thorgeirsson *et al*.^6^	2008	Spain	European ancestry	269	1474	Illumina 300	A/G
Thorgeirsson *et al*.^6^	2008	Netherland	European ancestry	90	2018	Illumina 300	A/G
Kohno *et al*.^10^	2011	Japan	East Asian	374	324	PCR	GA/GG
VanderWeele *et al*.^11^	2012	USA	European ancestry	1836	1452	Illumina 300 and TaqMan	AA/GA/GG
VanderWeele *et al*.^11^	2012	USA	European ancestry	2827	2345	Illumina 300	AA/GA/GG
VanderWeele *et al*.^11^	2012	IACR	European ancestry	1867	2463	Illumina 300	AA/GA/GG
VanderWeele *et al*.^11^	2012	Canada	European ancestry	333	501	Illumina 300	AA/GA/GG
Yang *et al*.^12^	2012	China	East Asian	1056	1061	PCR	GA/GG
Hansen *et al*.^28^	2010	USA	African	448	611	Illumina Golden Gate	A/G
Shiraishi *et al*.^7^	2009	Japan	East Asian	1250	936	TaqMan and the ABI Prism 7900HT	AA/GA/GG
He *et al*.^27^	2014	China	East Asian	301	318	Sequenom MassARRAY and PCR	A/G
Kaur-Knudsen *et al*.^24^	2012	CGPS	Europen ancestry	234	34347	TaqMan	AA/GA/GG
Ren *et al*.^13^	2013	China	East Asian	210	200	TaqMan	GA/GG
Liu *et al*.^25^	2010	GELCC	Europen ancestry	194	217	Illumina 550	A/G
Liu *et al*.^25^	2010	MCC	Europen ancestry	890	865	Illumina 550	A/G
Liu *et al*.^25^	2010	USA	Europen ancestry	1466	1389	Illumina 550	A/G
Liu *et al*.^25^	2010	USA	African	268	293	Illumina 550	A/G
Sakoda *et al*.^14^	2011	USA	Europen ancestry	745	1475	Illumina Golden Gate	AA/GA/GG
Amos *et al*.^15^	2010	USA	African	467	388	TaqMan	A/G
Kaur-Knudsen *et al*.^26^	2011	CCHS	Europen ancestry	308	9907	TaqMan	AA/GA/GG
Wassenaar *et al*.^16^	2013	USA	Europen ancestry	398	421	PCR	AA/GA and GG
Liu *et al*.^17^	2008	GELCC	Europen ancestry	178	204	SNP Array	AA/GA/GG
Landi *et al*.^18^	2009	NCI	Europen ancestry	13300	19666	SNP Array	A/G
All				33617	116639		

“AA/GA/GG” means the study have offered the data of genotypes AA/GA/GG both in cases and controls. “AA/GA and GG” means only the data of genotypes AA/GA and GG both in cases and controls have been offered in the study. “GA/GG” means only the data of genotypes GA/GG both in cases and controls have been offered in the study. “A/G” means only the data of genotypes A/G both in cases and controls have been offered in the study. All the alleles have been used A or G to express in our study to replace T or C in original articles.

IARC: International Agency for Research on Cancer; CGQS: Copenhagen General Population Study; GELCC: Caucasians of Genetic Epidemiology of Lung Cancer Consortium; MCC: Mayo Clinic Caucasians; NCI: National Cancer Institute.

**Table 2:** The result of heterogeneity test and meta-analysis in subgroup analysis.

Subgroup	Heterogeneity test			Meta-analysis		
	I^2^	P value	OR	95% CI	Z value	P value
All studies	39.10%	0.0161	1.303	[1.2682; 1.3388]	19.1601	<0.0001
European ancestry	18.40%	0.2296	1.2976	[1.2622; 1.3339]	18.473	<0.0001
African	42.20%	0.1586	1.5025	[1.2465; 1.8110]	4.2723	<0.0001
East Asian	66.90%	0.006	1.4759	[0.9957; 2.1878]	1.9383	0.0526
Japanese	0%	0.8387	2.2654	[1.5675; 3.2741]	4.3523	<0.0001
Chinese	56.50%	0.0753	1.1743	[0.7818; 1.7637]	0.774	0.4389
Removing the study of Wu *et al*. from Chinese	1.40%	0.3626	1.4235	[1.0146; 1.9972]	2.0437	0.041
Removing the study of Wu *et al*. from East Asian	12.4%	0.3356	1.7818	[1.3915; 2.2815]	4.5789	<0.0001
