# Supplementary material for: Analyzing large-scale samples confirms the association between the rs1051730 polymorphism and lung cancer susceptibility
Source: Sci Rep. 2015 Oct 28;5:15642. doi: 10.1038/srep15642 (PMC4623668; doi:10.1038/srep15642)
Supplement: Supplementary Information [file srep15642-s1.docx]

**Supporting Information**

**Analyzing large-scale samples confirms the association between the rs1051730 polymorphism and lung cancer susceptibility**

Zhijie Han^1^, Qinghua Jiang^1,^*, Tianjiao Zhang^2^, Xiaoliang Wu^2^, Rui Ma^2^, Jixuan Wang^3^, Yang Bai^2^, Rongjie Wang^2^, Renjie Tan^2^, Yadong Wang^2,^*

1 School of Life Science and Technology, Harbin Institute of Technology, Harbin, 150001, China.

2 School of Computer Science and Technology, Harbin Institute of Technology, Harbin, 150001, China.

3 School of Software, Harbin Institute of Technology, Harbin, 150001, China.

*Correspondence and requests for materials should be addressed to Qinghua Jiang ([qhjiang@hit.edu.cn](mailto:qhjiang@hit.edu.cn)) and Yadong Wang（[ydwang@hit.edu.cn](mailto:ydwang@hit.edu.cn)）.

**Table S1. The studies used for Meta-analysis in the allele model.**

| Study | Year | Country or Institution | Ethnicity | Allele A in case | Total allele A | Allele G in case | Total allele G | Kind of genotype |
| --- | --- | --- | --- | --- | --- | --- | --- | --- |
| Amos et al. | 2008 | UK | European ancestry | 1438 | 2042 | 2222 | 3530 | AA/GA/GG |
| Zienolddiny et al. | 2009 | Norway | European ancestry | 300 | 607 | 404 | 947 | AA/GA/GG |
| Schwartz et al. | 2009 | USA | European ancestry | 470 | 1091 | 694 | 1761 | AA/GA/GG |
| Schwartz et al. | 2009 | USA | African | 116 | 200 | 654 | 1434 | AA/GA/GG |
| Wu et al. | 2009 | China | East Asian | 63 | 139 | 2239 | 4463 | AA/GA/GG |
| Girard et al. | 2010 | USA | European ancestry | 33 | 72 | 61 | 117 | A/G |
| Girard et al. | 2010 | Japan | East Asian | 2 | 5 | 121 | 365 | A/G |
| Thorgeirsson et al. | 2008 | Iceland | European ancestry | 265 | 10098 | 400 | 19319 | A/G |
| Thorgeirsson et al. | 2008 | Spain | European ancestry | 130 | 705 | 139 | 1038 | A/G |
| Thorgeirsson et al. | 2008 | Netherland | European ancestry | 31 | 665 | 59 | 1443 | A/G |
| Kohno et al. | 2011 | Japan | East Asian | 25 | 35 | 723 | 1361 | GA/GG |
| VanderWeele et al. | 2012 | USA | European ancestry | 1542 | 2553 | 2130 | 4023 | AA/GA/GG |
| VanderWeele et al. | 2012 | USA | European ancestry | 2160 | 3768 | 3494 | 6576 | AA/GA/GG |
| VanderWeele et al. | 2012 | IACR | European ancestry | 1503 | 3183 | 2231 | 5477 | AA/GA/GG |
| VanderWeele et al. | 2012 | Canada | European ancestry | 265 | 608 | 401 | 1059 | AA/GA/GG |
| Yang et al. | 2012 | China | East Asian | 49 | 85 | 2063 | 4149 | GA/GG |
| Hansen et al. | 2010 | USA | African | 59 | 136 | 389 | 923 | A/G |
| Shiraishi et al. | 2009 | Japan | East Asian | 83 | 110 | 2417 | 4262 | AA/GA/GG |
| He et al. | 2014 | China | East Asian | 12 | 25 | 289 | 594 | A/G |
| Kaur-Knudsen et al. | 2012 | CGPS | European ancestry | 183 | 22583 | 285 | 46579 | AA/GA/GG |
| Ren et al. | 2013 | China | East Asian | 22 | 32 | 398 | 788 | GA/GG |
| Liu et al. | 2010 | GELCC | European ancestry | 79 | 143 | 115 | 268 | A/G |
| Liu et al. | 2010 | MCC | European ancestry | 354 | 670 | 536 | 1085 | A/G |
| Liu et al. | 2010 | USA | European ancestry | 552 | 1013 | 914 | 1842 | A/G |
| Liu et al. | 2010 | USA | African | 39 | 66 | 229 | 495 | A/G |
| Sakoda et al. | 2011 | USA | European ancestry | 607 | 1617 | 883 | 2823 | AA/GA/GG |
| Amos et al. | 2010 | USA | African | 78 | 116 | 389 | 739 | A/G |
| Kaur-Knudsen et al. | 2011 | CCHS | European ancestry | 246 | 6799 | 370 | 13631 | AA/GA/GG |
| Liu et al. | 2008 | GELCC | Europen ancestry | 151 | 270 | 205 | 494 | AA/GA/GG |
| Landi et al. | 2009 | NCI | Europen ancestry | 5493 | 12337 | 7807 | 20629 | A/G |

**Table S2. The studies used for Meta-analysis in the dominant model.**

| Study | Year | Country or Institution | Ethnicity | Genotype AA+GA in case | Total genotype AA+GA | Genotype GG in case | Total genotype GG | Kind of genotype |
| --- | --- | --- | --- | --- | --- | --- | --- | --- |
| Amos et al. | 2008 | UK | European ancestry | 1143 | 1654 | 687 | 1132 | AA/GA/GG |
| Zienolddiny et al. | 2009 | Norway | European ancestry | 242 | 493 | 110 | 284 | AA/GA/GG |
| Schwartz et al. | 2009 | USA | European ancestry | 375 | 875 | 207 | 551 | AA/GA/GG |
| Schwartz et al. | 2009 | USA | African | 106 | 185 | 279 | 632 | AA/GA/GG |
| Wu et al. | 2009 | China | East Asian | 62 | 138 | 1089 | 2163 | AA/GA/GG |
| Kohno et al. | 2011 | Japan | East Asian | 25 | 35 | 349 | 663 | GA/GG |
| VanderWeele et al. | 2012 | USA | European ancestry | 1195 | 2017 | 641 | 1271 | AA/GA/GG |
| VanderWeele et al. | 2012 | USA | European ancestry | 1736 | 3091 | 1091 | 2081 | AA/GA/GG |
| VanderWeele et al. | 2012 | IACR | European ancestry | 1193 | 2587 | 674 | 1743 | AA/GA/GG |
| VanderWeele et al. | 2012 | Canada | European ancestry | 210 | 498 | 123 | 336 | AA/GA/GG |
| Yang et al. | 2012 | China | East Asian | 49 | 85 | 1007 | 2032 | GA/GG |
| Shiraishi et al. | 2009 | Japan | East Asian | 80 | 106 | 1170 | 2080 | AA/GA/GG |
| Kaur-Knudsen et al. | 2012 | CGPS | Europen ancestry | 149 | 18955 | 85 | 15626 | AA/GA/GG |
| Ren et al. | 2013 | China | East Asian | 22 | 32 | 188 | 378 | GA/GG |
| Sakoda et al. | 2011 | USA | Europen ancestry | 490 | 1340 | 225 | 880 | AA/GA/GG |
| Kaur-Knudsen et al. | 2011 | CCHS | Europen ancestry | 196 | 5663 | 112 | 4552 | AA/GA/GG |
| Liu et al. | 2008 | GELCC | Europen ancestry | 151 | 270 | 205 | 494 | AA/GA/GG |

**Table S3. The studies used for Meta-analysis in the recessive model.**

| Study | Year | Country or Institution | Ethnicity | Genotype AA in case | Total genotype AA | Genotype GA+GG in case | Total genotype GA+GG | Kind of genotype |
| --- | --- | --- | --- | --- | --- | --- | --- | --- |
| Amos et al. | 2008 | UK | European ancestry | 295 | 388 | 1535 | 2398 | AA/GA/GG |
| Zienolddiny et al. | 2009 | Norway | European ancestry | 58 | 114 | 294 | 663 | AA/GA/GG |
| Schwartz et al. | 2009 | USA | European ancestry | 95 | 216 | 487 | 1210 | AA/GA/GG |
| Schwartz et al. | 2009 | USA | African | 10 | 15 | 375 | 802 | AA/GA/GG |
| VanderWeele et al. | 2012 | USA | European ancestry | 347 | 536 | 1489 | 2752 | AA/GA/GG |
| VanderWeele et al. | 2012 | USA | European ancestry | 424 | 677 | 2403 | 4495 | AA/GA/GG |
| VanderWeele et al. | 2012 | IACR | European ancestry | 310 | 596 | 1557 | 3734 | AA/GA/GG |
| VanderWeele et al. | 2012 | Canada | European ancestry | 55 | 110 | 279 | 723 | AA/GA/GG |
| Shiraishi et al. | 2009 | Japan | East Asian | 3 | 4 | 1247 | 2182 | AA/GA/GG |
| Kaur-Knudsen et al. | 2012 | CGPS | Europen ancestry | 34 | 3628 | 200 | 30953 | AA/GA/GG |
| Sakoda et al. | 2011 | USA | Europen ancestry | 117 | 277 | 628 | 1943 | AA/GA/GG |
| Kaur-Knudsen et al. | 2011 | CCHS | Europen ancestry | 50 | 1136 | 258 | 9079 | AA/GA/GG |
| Wassenaar et al. | 2013 | USA | Europen ancestry | 68 | 117 | 330 | 702 | AA and GA/GG |
| Liu et al. | 2008 | GELCC | Europen ancestry | 151 | 270 | 205 | 494 | AA/GA/GG |

**Table S4. The studies used for Meta-analysis in the additive model.**

| Study | Year | Country or Institution | Ethnicity | Genotype AA in case | Total genotype AA | Genotype GG in case | Total genotype GG | Kind of genotype |
| --- | --- | --- | --- | --- | --- | --- | --- | --- |
| Amos et al. | 2008 | UK | European ancestry | 295 | 388 | 687 | 1132 | AA/GA/GG |
| Zienolddiny et al. | 2009 | Norway | European ancestry | 58 | 114 | 110 | 284 | AA/GA/GG |
| Schwartz et al. | 2009 | USA | European ancestry | 95 | 216 | 207 | 551 | AA/GA/GG |
| Schwartz et al. | 2009 | USA | African | 10 | 15 | 279 | 632 | AA/GA/GG |
| VanderWeele et al. | 2012 | USA | European ancestry | 347 | 536 | 641 | 1271 | AA/GA/GG |
| VanderWeele et al. | 2012 | USA | European ancestry | 424 | 677 | 1091 | 2081 | AA/GA/GG |
| VanderWeele et al. | 2012 | IACR | European ancestry | 310 | 596 | 674 | 1743 | AA/GA/GG |
| VanderWeele et al. | 2012 | Canada | European ancestry | 55 | 110 | 123 | 336 | AA/GA/GG |
| Shiraishi et al. | 2009 | Japan | East Asian | 3 | 4 | 1170 | 2080 | AA/GA/GG |
| Kaur-Knudsen et al. | 2012 | CGPS | Europen ancestry | 34 | 3628 | 85 | 15626 | AA/GA/GG |
| Sakoda et al. | 2011 | USA | Europen ancestry | 117 | 277 | 225 | 880 | AA/GA/GG |
| Kaur-Knudsen et al. | 2011 | CCHS | Europen ancestry | 50 | 1136 | 112 | 4552 | AA/GA/GG |
| Liu et al. | 2008 | GELCC | Europen ancestry | 151 | 270 | 205 | 494 | AA/GA/GG |

**Table S5. The result of Sensitivity Analysis in allele model.**

| The study which is removed | Year | Country or Institution | Ethnicity | OR | 95% CI | P velue | Tau^2^ | I^2^ |
| --- | --- | --- | --- | --- | --- | --- | --- | --- |
| Amos et al. | 2008 | UK | European ancestry | 1.2976 | [1.2619; 1.3342] | < 0.0001 | 0.0044 | 39.10% |
| Zienolddiny et al. | 2009 | Norway | European ancestry | 1.3029 | [1.2677; 1.3389] | < 0.0001 | 0.0046 | 41.20% |
| Schwartz et al. | 2009 | USA | European ancestry | 1.3078 | [1.2723; 1.3443] | < 0.0001 | 0.0042 | 38.40% |
| Schwartz et al. | 2009 | USA | African | 1.3005 | [1.2656; 1.3363] | < 0.0001 | 0.004 | 38.10% |
| Wu et al. | 2009 | China | East Asian | 1.307 | [1.2719; 1.3429] | < 0.0001 | 0.0029 | 30.90% |
| Girard et al. | 2010 | USA | European ancestry | 1.3045 | [1.2696; 1.3403] | < 0.0001 | 0.0038 | 37.30% |
| Girard et al. | 2010 | Japan | East Asian | 1.3039 | [1.2682; 1.3388] | < 0.0001 | 0.0045 | 41.20% |
| Thorgeirsson et al. | 2008 | Iceland | European ancestry | 1.303 | [1.2685; 1.3402] | < 0.0001 | 0.0047 | 41.10% |
| Thorgeirsson et al. | 2008 | Spain | European ancestry | 1.3014 | [1.2665; 1.3373] | < 0.0001 | 0.0044 | 40.20% |
| Thorgeirsson et al. | 2008 | Netherland | European ancestry | 1.3036 | [1.2688; 1.3395] | < 0.0001 | 0.0044 | 40.80% |
| Kohno et al. | 2011 | Japan | East Asian | 1.302 | [1.2672; 1.3378] | < 0.0001 | 0.0041 | 38.70% |
| VanderWeele et al. | 2012 | USA | European ancestry | 1.299 | [1.2630; 1.3360] | < 0.0001 | 0.0048 | 40.40% |
| VanderWeele et al. | 2012 | USA | European ancestry | 1.319 | [1.2816; 1.3574] | < 0.0001 | 0.0037 | 32.80% |
| VanderWeele et al. | 2012 | IACR | European ancestry | 1.3032 | [1.2666; 1.3408] | < 0.0001 | 0.0051 | 41.20% |
| VanderWeele et al. | 2012 | Canada | European ancestry | 1.3037 | [1.2686; 1.3398] | < 0.0001 | 0.0046 | 41.10% |
| Yang et al. | 2012 | China | East Asian | 1.3028 | [1.2679; 1.3386] | < 0.0001 | 0.0045 | 41.10% |
| Hansen et al. | 2010 | USA | African | 1.3046 | [1.2697; 1.3405] | < 0.0001 | 0.0042 | 39.50% |
| Shiraishi et al. | 2009 | Japan | East Asian | 1.2996 | [1.2648; 1.3354] | < 0.0001 | 0.0029 | 31.10% |
| He et al. | 2014 | China | East Asian | 1.3035 | [1.2686; 1.3393] | < 0.0001 | 0.0044 | 40.60% |
| Kaur-Knudsen et al. | 2012 | CGPS | Europen ancestry | 1.3025 | [1.2674; 1.3387] | < 0.0001 | 0.0046 | 41.10% |
| Ren et al. | 2013 | China | East Asian | 1.3021 | [1.2673; 1.3379] | < 0.0001 | 0.0041 | 39.00% |
| Liu et al. | 2010 | GELCC | Europen ancestry | 1.3017 | [1.2669; 1.3375] | < 0.0001 | 0.0042 | 39.60% |
| Liu et al. | 2010 | MCC | Europen ancestry | 1.3064 | [1.2712; 1.3426] | < 0.0001 | 0.0042 | 39.00% |
| Liu et al. | 2010 | USA | Europen ancestry | 1.306 | [1.2705; 1.3424] | < 0.0001 | 0.0045 | 40.20% |
| Liu et al. | 2010 | USA | African | 1.3021 | [1.2673; 1.3379] | < 0.0001 | 0.0043 | 40.00% |
| Sakoda et al. | 2011 | USA | Europen ancestry | 1.3022 | [1.2667; 1.3388] | < 0.0001 | 0.0048 | 41.10% |
| Amos et al. | 2010 | USA | African | 1.301 | [1.2661; 1.3368] | < 0.0001 | 0.0039 | 37.60% |
| Kaur-Knudsen et al. | 2011 | CCHS | Europen ancestry | 1.3019 | [1.2667; 1.3381] | < 0.0001 | 0.0046 | 41.00% |
| Liu et al. | 2008 | GELCC | Europen ancestry | 1.2996 | [1.2648; 1.3354] | < 0.0001 | 0.0035 | 35.30% |
| Landi et al. | 2009 | NCI | Europen ancestry | 1.2947 | [1.2517; 1.3392] | < 0.0001 | 0.0063 | 40.70% |
| Pooled estimate |  |  |  | 1.303 | [1.2682; 1.3388] | < 0.0001 | 0.0043 | 39.10% |

**Table S6. The result of Sensitivity Analysis in dominant model.**

| The study which is removed | Year | Country or Institution | Ethnicity | OR | 95% CI | P velue | Tau^2^ | I^2^ |
| --- | --- | --- | --- | --- | --- | --- | --- | --- |
| Amos | 2008 | UK | European ancestry | 1.4145 | [1.2827; 1.5598] | < 0.0001 | 0.0188 | 58.40% |
| Zienolddiny | 2009 | Norway | European ancestry | 1.4097 | [1.2840; 1.5478] | < 0.0001 | 0.0112 | 58.50% |
| Schwartz | 2009 | USA | European ancestry | 1.4304 | [1.3008; 1.5729] | < 0.0001 | 0.0176 | 58.20% |
| Schwartz | 2009 | USA | African | 1.4019 | [1.2795; 1.5361] | < 0.0001 | 0.0172 | 57.10% |
| Wu | 2009 | China | East Asian | 1.4378 | [1.2816; 1.5367] | < 0.0001 | 0.01 | 45.30% |
| Kohno | 2011 | Japan | East Asian | 1.4056 | [1.2857; 1.5366] | < 0.0001 | 0.0157 | 57.10% |
| VanderWeele | 2012 | USA | European ancestry | 1.4172 | [1.2834; 1.5649] | < 0.0001 | 0.0195 | 58.60% |
| VanderWeele | 2012 | USA | European ancestry | 1.444 | [1.3249; 1.5739] | < 0.0001 | 0.0162 | 42.90% |
| VanderWeele | 2012 | IACR | European ancestry | 1.4264 | [1.2891; 1.5783] | < 0.0001 | 0.0208 | 59.10% |
| VanderWeele | 2012 | Canada | European ancestry | 1.4257 | [1.2979; 1.5660] | < 0.0001 | 0.0174 | 56.40% |
| Yang | 2012 | China | East Asian | 1.4168 | [1.2916; 1.5540] | < 0.0001 | 0.0173 | 59.10% |
| Shiraishi | 2009 | Japan | East Asian | 1.3886 | [1.2763; 1.5107] | < 0.0001 | 0.0125 | 51.10% |
| Kaur-Knudsen | 2012 | CGPS | Europen ancestry | 1.4141 | [1.2867; 1.5542] | < 0.0001 | 0.0176 | 58.90% |
| Ren | 2013 | China | East Asian | 1.4064 | [1.2862; 1.5378] | < 0.0001 | 0.0159 | 57.30% |
| Sakoda | 2011 | USA | Europen ancestry | 1.395 | [1.2706; 1.5218] | < 0.0001 | 0.0144 | 52.70% |
| Kaur-Knudsen | 2011 | CCHS | Europen ancestry | 1.4162 | [1.2875; 1.5578] | < 0.0001 | 0.0179 | 59.00% |
| Liu et al. | 2008 | GELCC | Europen ancestry | 1.4034 | [1.2937; 1.5466] | < 0.0001 | 0.0161 | 57.40% |
| Pooled estimate |  |  |  | 1.4145 | [1.3271; 1.5578] | < 0.0001 | 0.0161 | 58.70% |

**Table S7. The result of Sensitivity Analysis in recessive model.**

| The study which is removed | Year | Country or Institution | Ethnicity | OR | 95% CI | P velue | Tau^2^ | I^2^ |
| --- | --- | --- | --- | --- | --- | --- | --- | --- |
| Amos | 2008 | UK | European ancestry | 1.504 | [1.3909; 1.6263] | < 0.0001 | 0 | 0.00% |
| Zienolddiny | 2009 | Norway | European ancestry | 1.5387 | [1.4013; 1.6281] | < 0.0001 | 0.0012 | 5.60% |
| Schwartz | 2009 | USA | European ancestry | 1.5587 | [1.4431; 1.6836] | < 0.0001 | 0 | 0.00% |
| Schwartz | 2009 | USA | African | 1.5265 | [1.4167; 1.6449] | < 0.0001 | 0.0014 | 6.40% |
| VanderWeele | 2012 | USA | European ancestry | 1.5246 | [1.4063; 1.6529] | < 0.0001 | 0.0026 | 2.70% |
| VanderWeele | 2012 | USA | European ancestry | 1.5484 | [1.4248; 1.6828] | < 0.0001 | 0.0021 | 7.80% |
| VanderWeele | 2012 | IACR | European ancestry | 1.5328 | [1.4115; 1.6645] | < 0.0001 | 0.0028 | 10.10% |
| VanderWeele | 2012 | Canada | European ancestry | 1.5275 | [1.4161; 1.6478] | < 0.0001 | 0.0023 | 9.80% |
| Shiraishi | 2009 | Japan | East Asian | 1.5289 | [1.4191; 1.6472] | < 0.0001 | 0.0021 | 9.40% |
| Kaur-Knudsen | 2012 | CGPS | Europen ancestry | 1.5325 | [1.4203; 1.6536] | < 0.0001 | 0 | 9.70% |
| Sakoda | 2011 | USA | Europen ancestry | 1.5295 | [1.4150; 1.6532] | < 0.0001 | 0.0025 | 10.10% |
| Kaur-Knudsen | 2011 | CCHS | Europen ancestry | 1.5273 | [1.4146; 1.6490] | < 0.0001 | 0.0023 | 9.90% |
| Wassenaar | 2013 | USA | Europen ancestry | 1.5284 | [1.4168; 1.6487] | < 0.0001 | 0.0023 | 10.00% |
| Liu et al. | 2008 | GELCC | Europen ancestry | 1.5105 | [1.4199; 1.6479] | < 0.0001 | 0.0022 | 0.00% |
| Pooled estimate |  |  |  | 1.5296 | [1.4263; 1.6599] | < 0.0001 | 0.0006 | 9.80% |

**Table S8. The result of Sensitivity Analysis in additive model.**

| The study which is removed | Year | Country or Institution | Ethnicity | OR | 95% CI | P velue | Tau^2^ | I^2^ |
| --- | --- | --- | --- | --- | --- | --- | --- | --- |
| Amos | 2008 | UK | European ancestry | 1.7164 | [1.5742; 1.8713] | < 0.0001 | 0.0084 | 24.90% |
| Zienolddiny | 2009 | Norway | European ancestry | 1.7536 | [1.6131; 1.9063] | < 0.0001 | 0.011 | 22.70% |
| Schwartz | 2009 | USA | European ancestry | 1.786 | [1.6406; 1.9443] | < 0.0001 | 0.004 | 14.20% |
| Schwartz | 2009 | USA | African | 1.7455 | [1.6077; 1.8951] | < 0.0001 | 0.0098 | 30.50% |
| VanderWeele | 2012 | USA | European ancestry | 1.7393 | [1.5909; 1.9015] | < 0.0001 | 0.0127 | 31.90% |
| VanderWeele | 2012 | USA | European ancestry | 1.8189 | [1.6585; 1.9948] | < 0.0001 | 0.006 | 17.30% |
| VanderWeele | 2012 | IACR | European ancestry | 1.7566 | [1.6035; 1.9244] | < 0.0001 | 0.0134 | 32.20% |
| VanderWeele | 2012 | Canada | European ancestry | 1.7501 | [1.6099; 1.9025] | < 0.0001 | 0.0112 | 32.40% |
| Shiraishi | 2009 | Japan | East Asian | 1.7487 | [1.6117; 1.8989] | < 0.0001 | 0.0106 | 32.10% |
| Kaur-Knudsen | 2012 | CGPS | Europen ancestry | 1.7502 | [1.6096; 1.9031] | < 0.0001 | 0.0113 | 32.40% |
| Sakoda | 2011 | USA | Europen ancestry | 1.7198 | [1.5787; 1.8736] | < 0.0001 | 0.0087 | 0.00% |
| Kaur-Knudsen | 2011 | CCHS | Europen ancestry | 1.7455 | [1.6041; 1.8993] | < 0.0001 | 0.0114 | 32.10% |
| Liu et al. | 2008 | GELCC | Europen ancestry | 1.7494 | [1.5885; 1.8740] | < 0.0001 | 0 | 26.30% |
| Pooled estimate |  |  |  | 1.7254 | [1.6109; 1.8982] | < 0.0001 | 0.0073 | 32.00% |

**
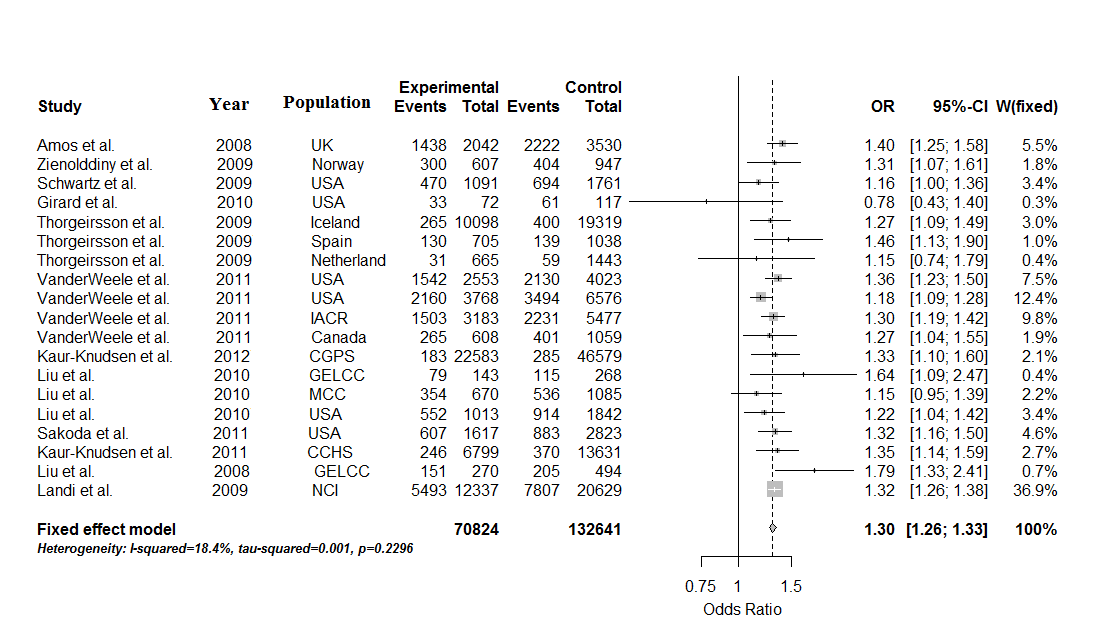
**

**Figure S1. The result of meta-analysis in European ancestry population by using the allele model**

**
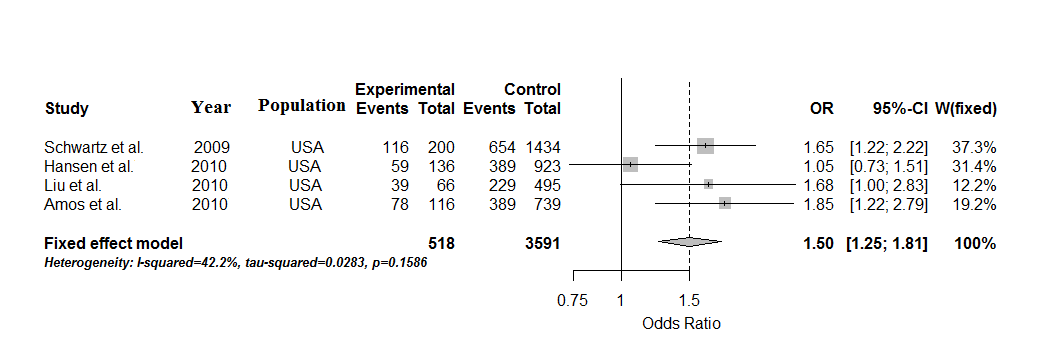
**

**Figure S2. The result of meta-analysis in African population by using the allele model**

**
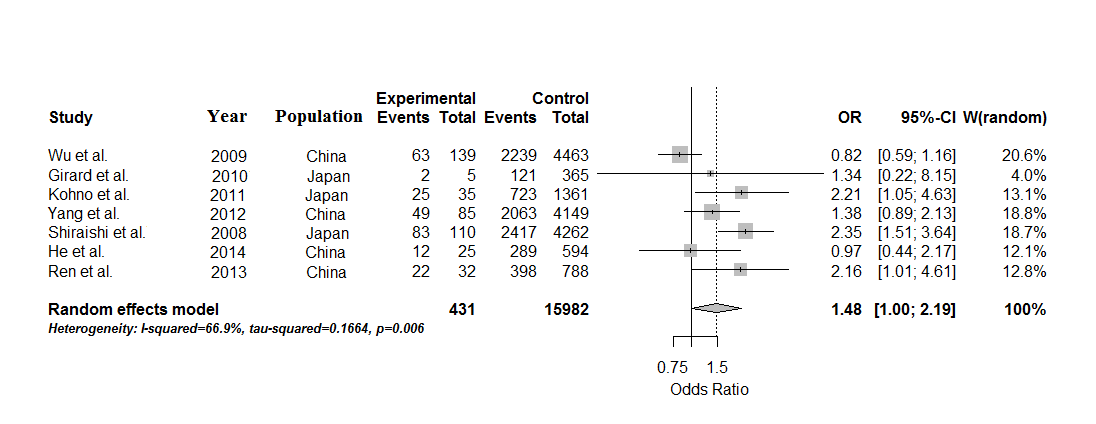
**

**Figure S3. The result of meta-analysis in East Asian population by using the allele model**


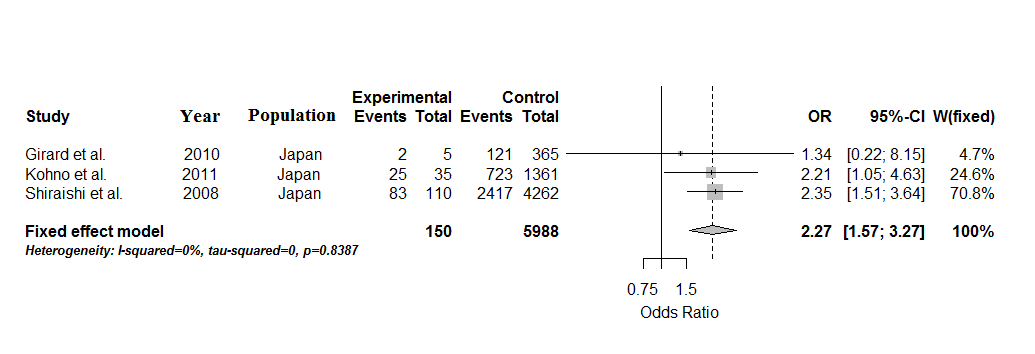


**Figure S4. The result of meta-analysis in Japanese population by using the allele model**

**
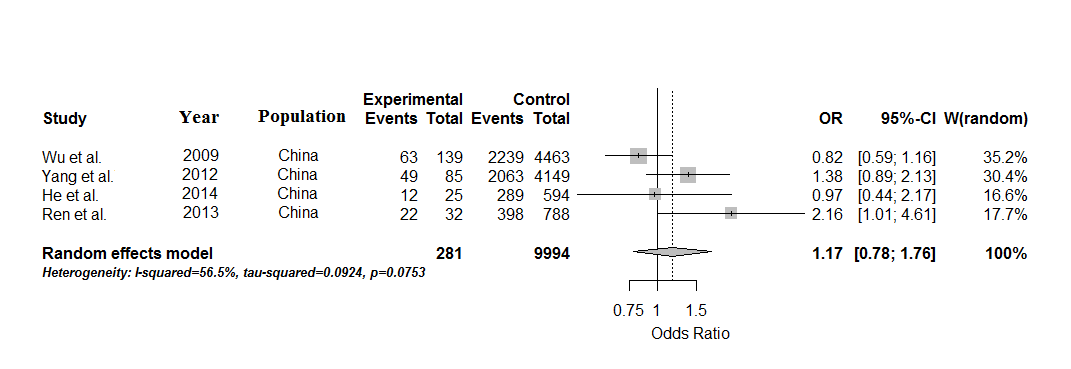
**

**Figure S5. The result of meta-analysis in Chinese population by using the allele model**


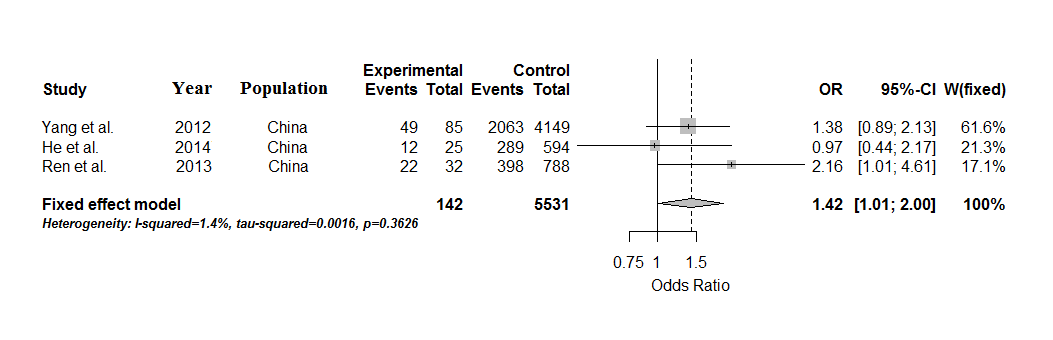


**Figure S6. The result of meta-analysis in the subgroup removed Wu’ study from Chinese population**

**
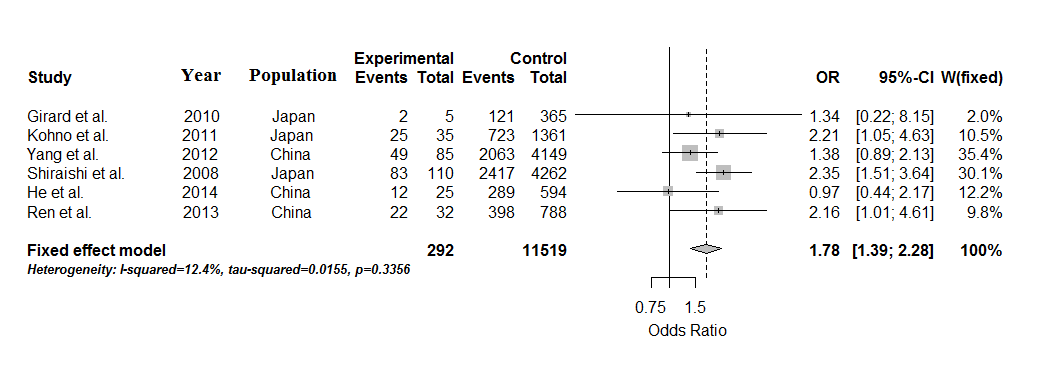
**

**Figure S7. The result of meta-analysis in the subgroup removed Wu’ study from East Asian population**
